# An Elastoplastic Constitutive Model for Steel Slag Aggregate Concrete Under Multiaxial Stress States Based on Non-Uniform Hardening Theory

**DOI:** 10.3390/ma18174124

**Published:** 2025-09-02

**Authors:** Zhijun Chen, Liang Huang, Yiwei Yang, Teng Dong

**Affiliations:** 1 School of Civil and Architectural Engineering, Hunan Institute of Technology, Hengyang 421002, China; 2 College of Civil Engineering, Hunan University, Changsha 410082, China

**Keywords:** steel slag aggregate concrete, yield criterion, nonassociative flow rule, constitutive model

## Abstract

Steel slag aggregate concrete (SAC) is widely recognized as a high-performance and sustainable construction material. However, its broader structural application has been impeded by the limited development of reliable constitutive models. Building upon the well-established non-uniform hardening plasticity theory, this study proposes a comprehensive theoretical framework to establish a stress–strain relationship model for SAC under complex stress states. To this end, a multiaxial elastoplastic constitutive model for SAC is developed through the following steps: (1) The Guo–Wang failure criterion is employed as the bounding surface, from which a yield criterion is formulated to capture the characteristic mechanical responses of SAC under multiaxial loading; (2) Based on fundamental plasticity theory, the stress–strain relationship is derived by integrating the proposed yield function with a non-associated flow rule using a Drucker–Prager-type plastic potential function, while ensuring consistency conditions are satisfied; (3) A parameter calibration methodology is introduced and applied using experimental data from uniaxial and multiaxial tests on SAC; (4) A numerical implementation scheme is developed in MATLAB 2024a, and the model is validated through computational simulations. The validation results confirm that the proposed model reliably captures the stress–strain behavior of SAC under complex loading conditions. Overall, this study not only delivers a robust multiaxial constitutive model for SAC, but also offers a systematic modeling approach that may serve as a reference for the further development of constitutive theories for steel slag-based concretes and their broader application in structural engineering.

## 1. Introduction

The burgeoning global accumulation of steel slag has created an urgent need for effective and high-value recycling strategies [1]. Among the various approaches investigated for reutilizing steel slag [2,3,4,5,6,7], its use as aggregate in concrete production has emerged as one of the most promising solutions [8,9,10].

The intrinsic properties of steel slag make it a suitable alternative to natural aggregates. First, compared to natural aggregates, steel slag particles exhibit a rougher surface texture and more angular geometry [11], which enhances mechanical interlocking and improves the quality of the interfacial transition zone (ITZ) between the aggregate and the cementitious matrix [12,13,14]. Second, steel slag contains cementitious mineral phases such as C_2_S, C_3_S, and C_4_AF, which contribute to the development of stronger bonding at the aggregate–paste interface [15]. Third, steel slag aggregate (SA) demonstrates superior mechanical properties, including lower crushing indices [16], reduced impact values [17], and lower Los Angeles abrasion losses [18], all of which underscore its potential as a high-performance and environmentally friendly aggregate for the concrete industry [19].

Owing to the unique physicochemical characteristics of steel slag aggregate, concrete incorporating this material exhibits performance advantages across multiple dimensions. Previous studies [20,21,22,23] have shown that steel slag aggregate concrete (SAC) achieves compressive strength improvements of 0–30%, tensile strength gains of 10–60%, and flexural strength enhancements of 15–70% compared to conventional concrete, demonstrating superior mechanical performance. Additionally, SAC generally offers comparable or improved durability, including better resistance to permeability [24], carbonation [25], acid/alkali corrosion [26], and freeze–thaw cycles [27]. These favorable properties highlight SAC’s potential as a sustainable alternative for structural applications. Notably, SAC has also demonstrated enhanced radiation shielding capabilities compared to ordinary concrete [9], with performance approaching that of specialized materials such as barytes concrete [28], suggesting its applicability in complex infrastructure such as nuclear containment vessels.

The application of SAC in both conventional and specialized structural systems necessitates rigorous evaluation of structural safety and economic feasibility. Such assessments depend critically on accurate constitutive models to predict the material’s load-bearing capacity and deformation behavior. Despite growing interest, research on the constitutive behavior of SAC remains limited and lags behind the practical needs of engineering applications. A few studies have investigated SAC’s mechanical behavior and failure characteristics under uniaxial compression and proposed corresponding stress–strain models [20,29]. In addition, simplified failure criteria have been formulated based on multiaxial test data [30,31]. However, the development of comprehensive multiaxial constitutive models for SAC still requires substantial experimental and theoretical advancements. Recent progress on triaxial compression testing of cementitious composites, such as 3D-printed UHPC and coal-reject concrete, further highlights the importance of developing robust constitutive models in structural application [32,33].

In our earlier work [34], a practical and scalable method for stabilizing the volumetric expansion of steel slag was developed, thereby addressing the key barrier to its structural application. Concurrently, the feasibility of replacing natural aggregates with steel slag aggregate in concrete production was demonstrated. Building on this foundation, a subsequent study [35] conducted a full suite of multiaxial mechanical tests on SAC, revealing its failure mechanisms, multiaxial strength evolution, and ultimate compressive deformation characteristics under complex loading. These experiments also yielded complete stress–strain curves under multiaxial stress states. More importantly, a SAC-specific failure criterion was successfully proposed, enabling accurate prediction of SAC strength under arbitrary stress conditions. These achievements naturally raise a fundamental question: can the deformation behavior of SAC under general stress states also be reliably predicted? In other words, is it possible to construct a robust constitutive model tailored to SAC?

Taken together, the findings of our previous investigations [34,35] provide a solid experimental and theoretical foundation for developing a multiaxial constitutive model for SAC. Within this framework, the present study aims to formulate an elastoplastic stress–strain model for SAC by integrating the established failure criterion with classical plasticity theory. Model parameters are calibrated using the existing multiaxial experimental data, and the model’s predictive capability is subsequently verified through numerical simulation. This work contributes to the advancement of constitutive modeling for SAC and provides the theoretical basis necessary to support its broader adoption in structural concrete applications.

## 2. Form of Incremental Constitutive Relation

### 2.1. Incremental Form of Stress–Strain Relationship for SAC

Since our previous study [35] revealed that SAC specimens exhibit pronounced nonlinear deformation once the applied uniaxial compressive stress exceeds 50% of the peak strength (i.e., 0.5 *f*_c_), it is reasonable to assume that the total strain increment ($d\varepsilon_{ij}$) under external loading consists of both the elastic strain increment ($d\varepsilon_{ij}^{e}$) and the plastic strain increment ($d\varepsilon_{ij}^{p}$).

(1) $$d\varepsilon_{ij}=d\varepsilon_{ij}^{e}+d\varepsilon_{ij}^{p}$$

According to the generalized Hooke’s law, the stress increment ($d\sigma_{ij}$) of SAC sample can be expressed as:

(2) $$d\sigma_{ij}=C_{ijkl}d\varepsilon_{kl}^{e}=C_{ijkl}(d\varepsilon_{kl}-d\varepsilon_{kl}^{p})$$

where *C_ijkl_* is the elastic stiffness tensor of SAC.

The consistency condition in plasticity theory requires that, during plastic deformation, the stress state must remain on the yield surface. Accordingly, the following condition must be satisfied:

(3) $$df=\frac{\partial f}{\partial \sigma_{ij}}d\sigma_{ij}+\frac{\partial f}{\partial \tau }\frac{\partial \tau }{\partial \varepsilon_{p}}d\varepsilon_{p}=0$$

where *f* = 0 defines the yield surface equation of SAC, and its explicit mathematical form will be presented in a later section. In this expression, *τ* denotes the effective stress and *ε_p_* represents the effective plastic strain. These two quantities are related by the following expression:

(4) $$\frac{d\tau }{d\varepsilon_{p}}=H^{p}$$

where $H^{p}$ is the plastic modulus of SAC. The specific functional form $H^{p}$ will be determined based on the calibration results derived from uniaxial and multiaxial mechanical performance tests.

By substituting Equations (2) and (4) into Equation (3), the following expression can be derived:

(5) $$\frac{\partial f}{\partial \sigma_{ij}}C_{ijkl}(d\varepsilon_{kl}-d\varepsilon_{kl}^{p})+\frac{\partial f}{\partial \tau }H^{p}d\varepsilon_{p}=0$$

The plastic strain increment $d\varepsilon_{kl}^{p}$ in Equation (5) can be explicitly expressed based on the plastic flow rule as follows:

(6) $$d\varepsilon_{kl}^{p}=d\lambda \frac{\partial g}{\partial \sigma_{kl}}$$

where d*λ* is a non-negative scalar known as the plastic multiplier, which determines the magnitude of the plastic strain increment in SAC. Its value can be obtained by enforcing the consistency condition defined in Equation (3). The function *g* denotes the plastic potential of SAC, and the gradient $\partial g/\partial \sigma_{kl}$ defines the direction of the plastic flow in stress space.

According to classical plasticity theory [36], the effective plastic strain *ε_p_* of SAC, as introduced in Equation (5), can be defined by the following expression:

(7) $$d\varepsilon_{p}=C\sqrt{d\varepsilon_{ij}^{p}d\varepsilon_{ij}^{p}}=C\sqrt{\frac{\partial g}{\partial \sigma_{ij}}\frac{\partial g}{\partial \sigma_{ij}}}d\lambda =\phi d\lambda$$

where *C* is a positive constant that can be determined through simple experimental calibration. The term *ϕ* is a scalar function dependent on the current stress state of SAC, and its explicit form will be provided in a subsequent section.

By substituting Equations (6) and (7) into Equation (5), the plastic multiplier d*λ* can be explicitly determined based on the consistency condition. The resulting expression for d*λ* is derived as follows:

(8) $$d\lambda =\frac{1}{h}\frac{\partial f}{\partial \sigma_{pq}}C_{pqkl}d\varepsilon_{kl}$$

where

(9) $$h=\frac{\partial f}{\partial \sigma_{mn}}C_{mnpq}\frac{\partial g}{\partial \sigma_{pq}}-H^{p}\frac{\partial f}{\partial \tau }\phi$$

where $\frac{\partial f}{\partial \tau }$ represents the partial derivative of the yield surface of SAC with respect to the effective stress.

By substituting Equations (6) and (8) into Equation (2), the incremental form of the elastoplastic multiaxial stress–strain relationship for SAC can be derived as follows:

(10) $$d\sigma_{ij}=(C_{ijkl}+C_{ijkl}^{p})d\varepsilon_{kl}$$

where $C_{ijkl}^{p}$ denotes the plastic stiffness tensor of SAC, and its explicit form is given by:

(11) $$C_{ijkl}^{p}=-\frac{1}{h}H_{ij}^{*}H_{kl}$$

where

(12) $$H_{ij}^{*}=C_{ijmn}\frac{\partial g}{\partial \sigma_{mn}}$$

(13) $$H_{kl}=\frac{\partial f}{\partial \sigma_{pq}}C_{pqkl}$$

The above derivation demonstrates that, once the yield function *f* and plastic potential function *g* for SAC are explicitly defined, and the expressions for $H^{p}$, ∂f/∂τ and *ϕ* in Equation (9) are determined based on the experimental data from our previous studies [34,35], the elastoplastic constitutive model of SAC can then be fully formulated through Equation (10).

### 2.2. Flow Rule

Experimental results [34,35] have shown that under compressive loading—whether uniaxial or multiaxial—the volumetric strain *ε*_v_ of SAC specimens decreases progressively until the minimum principal stress *σ*_3_ reaches approximately 80% of the ultimate compressive strength. Beyond this threshold, further increases in compressive stress lead to a reversal, with the volumetric strain beginning to increase. Given that hydrostatic pressure generally induces volumetric contraction, the observed transition from initial compaction to subsequent dilation implies that SAC exhibits a volumetric dilatancy effect. In particular, it suggests that deviatoric stress also contributes to volume expansion in SAC.

This dilatant behavior is inconsistent with the predictions of an associated flow rule, which assumes that plastic strain increments are normal to the yield surface. As such, a non-associated flow rule is required to accurately capture both the magnitude and direction of the plastic strain increment in SAC. In this study, the well-known Drucker–Prager model is adopted as the plastic potential function for SAC. The plastic potential function of SAC based on the Drucker–Prager model is defined as follows:

(14) $$g(\sigma_{ij},\alpha_{p})=\alpha_{p}I_{1}+\sqrt{J_{2}}$$

where *α_p_* is a material parameter governing the dilatancy of SAC, *I*_1_ is the first invariant of the stress tensor, and *J*_2_ is the second invariant of the deviatoric stress tensor.

Accordingly, the plastic strain increment can be expressed as

(15) $$d\varepsilon_{ij}^{p}=d\lambda \frac{\partial g}{\partial \sigma_{ij}}=d\lambda (\alpha_{p}\delta_{ij}+\frac{s_{ij}}{2\sqrt{J_{2}}})$$

According to Equation (15), the corresponding volumetric plastic strain increment becomes

(16) $$d\varepsilon_{v}^{p}=d\varepsilon_{\mathrm{ii}}^{p}=3\alpha_{p}d\lambda$$

Equation (16) indicates that the parameter *α_p_* assumes a negative value during the initial yielding stage of SAC, reflecting volumetric contraction, and gradually transitions to a positive value as the material approaches failure, corresponding to volumetric dilation. The detailed methodology for determining the value of *α_p_* will be presented in Section 3 of this paper.

### 2.3. Failure Surface and Yield Criterion

In our previous study [34], it was demonstrated that the Guo–Wang-based failure criterion not only captures the key geometric features commonly observed in the failure surface of concrete, but also yields accurate predictions of the multiaxial ultimate strength of SAC. Therefore, the Guo–Wang model was adopted in this study to define the failure surface function of SAC. The corresponding failure function can be expressed as

(17) $$F(r,\sigma_{m},\theta )=r-r_{f}(\sigma_{m},\theta )=0$$

where *r* denotes the length of the deviatoric part of the stress tensor; *σ_m_* is the mean normal stress, and *θ* represents the Lode angle.

The algebraic expression of $r_{f}(\sigma_{m},\theta )$ is given as follows:

(18) $$r_{f}(\sigma_{m},\theta )=\sqrt{3}af_{c}{\left(\frac{b-\frac{\sigma_{m}}{f_{c}}}{c(\theta )-\frac{\sigma_{m}}{f_{c}}}\right)}^{d}$$

where the function *c*(*θ*) is defined as

(19) $$c(\theta )=c_{t}{\left(\cos1.5\theta \right)}^{\alpha }+c_{c}{\left(\sin1.5\theta \right)}^{\beta }$$

**Figure 1:**
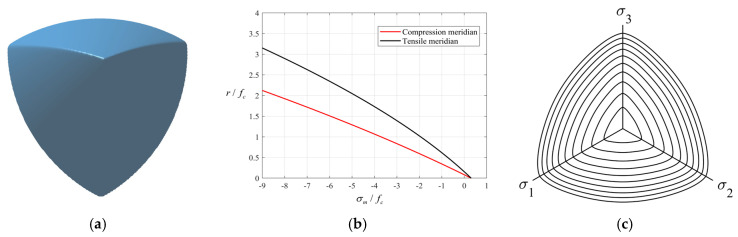
SAC failure surface based on Guo–Wang criterion [34]. (**a**) Failure surface; (**b**) tensile and compressive meridians; (**c**) trace on the deviatoric plane.

In these expressions, *f*_c_ denotes the peak uniaxial compressive strength of the SAC specimen. The parameters *a*, *b*, *c_t_*, *c_c_*, *d*, *α* and *β* are determined from the pre-established failure criterion for SAC proposed in reference [34], and their values are listed in Table 1. The corresponding failure surface of SAC based on the Guo–Wang model is illustrated in Figure 1.

To better elucidate the formulation of the SAC yield criterion in this study, it is necessary to first provide a brief introduction to the Non-Uniform Hardening Theory. The term ‘Non-Uniform Hardening Theory’ refers to the constitutive framework developed by W.F. Chen and D.J. Han [37,38], in which the yield surfaces evolve in a non-uniform fashion—the shape of the loading surface changes (rather than simply expanding isotropically) and the hardening modulus depends on both mean stress and Lode angle. This model captures key inelastic behaviors of concrete, including brittle tensile failure, ductile compression response, hydrostatic pressure sensitivity, and volumetric dilation under confinement—features that align precisely with the multiaxial behavior exhibited by steel slag aggregate concrete (SAC). By adopting this framework, our model can achieve a more accurate representation of SAC’s complex stress–strain response under multiaxial loading.

During the multiaxial mechanical testing of SAC [34], it was observed that the plastic deformation capacity and ultimate strength of SAC are primarily governed by the level of lateral confinement (i.e., cylindrical pressure) applied to the specimen. Additionally, the material response exhibits sensitivity to the Lode angle (i.e., the value of *σ*_2_/*σ*_3_). Given that the cylindrical pressure closely approximates the mean stress, it is reasonable to assume that the confinement-induced hardening behavior of SAC is predominantly controlled by its mean stress *σ_m_* and Lode angle *θ*. Based on these observations, and to accurately describe the yield behavior of SAC as a function of mean stress and Lode angle, the yield criterion proposed by Chen [39] is introduced in this study to define the material’s yield surface. The yield function adopted in this study is defined as

(20) $$f=r-k(k_{0},\sigma_{m})r_{f}(\sigma_{m},\theta )=r-kr_{f}=0$$

where *k*(*k*_0_,*σ_m_*) is a scaling function that reflects the strength evolution of SAC during loading, and *k*_0_ is a hardening parameter that quantifies the degree of hardening. The parameter *k*_0_ lies within the range *k_y_* ≤ *k*_0_ ≤ 1, where (1) *k*_0_ = *k_y_* corresponds to the initial yield surface of SAC; (2) *k*_0_ = 1 corresponds to the failure surface, indicating that the current loading surface has reached the ultimate strength envelope.

**Figure 2:**
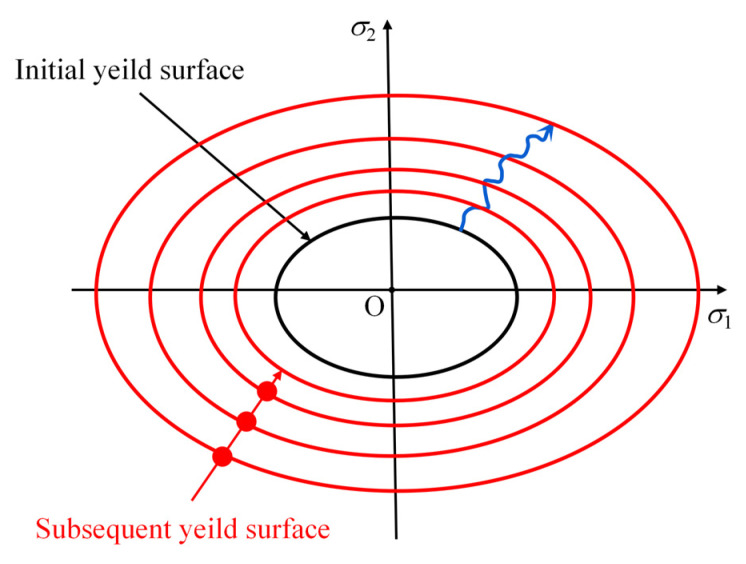
SAC failure surface based on Guo–Wang criterion.

The loading surface defined by Equation (20) retains a consistent shape on the deviatoric plane (see Figure 2.), while its size scales proportionally with changes in the hardening function *k*(*k*_0_,*σ_m_*). As a result, the model conforms to the isotropic hardening rule in the deviatoric plane.

**Figure 3:**
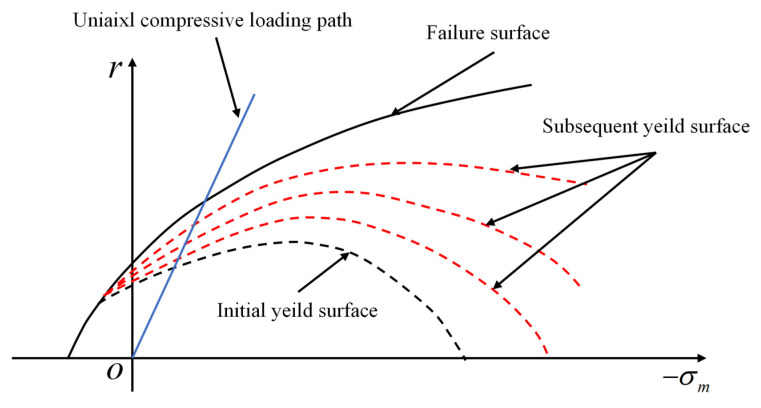
Yield surface model of SAC [38].

In contrast, the evolution of the yield surface on the hydrostatic plane exhibits a different behavior, as illustrated in Figure 3. The surface initially appears as a closed boundary but progressively opens in a non-uniform manner under increasing hydrostatic pressure, thereby capturing the hydrostatic pressure sensitivity characteristic of concrete. This indicates that, within the present constitutive framework, the yield surface of SAC does not follow an isotropic hardening rule in the hydrostatic plane.

It is worth emphasizing that, as observed in the SAC multiaxial mechanical performance tests [34], the material exhibits significantly different mechanical responses under varying stress states. Specifically, SAC specimens fail in a brittle manner under tensile loading, exhibit enhanced ductility under compressive stress, and demonstrate clear sensitivity to static hydrostatic pressure. These distinct behaviors are consistent with the general characteristics of plain concrete and must be accurately represented by the SAC yield criterion defined in Equation (20).

According to Equation (20), the size and shape of the SAC yield surface are governed by the hardening function *k* and the failure surface *r_f_*. Consequently, a properly formulated hardening function *k* must be capable of capturing the stress-path-dependent hardening behavior exhibited in different stress regions.

**Figure 4:**
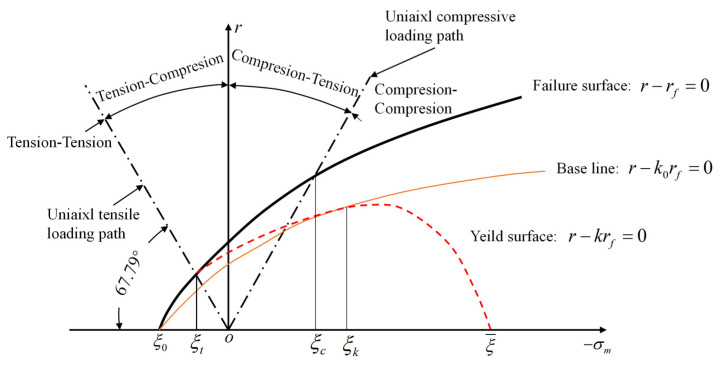
Yield surface of concrete [38].

To address this, Han and Chen [37,38] proposed partitioning both the initial and subsequent yield surfaces of concrete into four distinct regions: tension–tension, tension–compression, compression–tension, and compression–compression (Figure 4). Based on the characteristic mechanical responses within each region, they developed four separate expressions for the hardening function *k*, as detailed in Equations (21) and (22).

(21) $$k(k_{0},\sigma_{m})=\left\{\begin{array}{ll} 1 & \;(\sigma_{m}\geq \xi_{t})\\ k_{1}(\sigma_{m}) & \;(\xi_{t}>\sigma_{m}\geq \xi_{c})\\ k_{0} & \;(\xi_{c}>\sigma_{m}\geq \xi_{k})\\ k_{2}(\sigma_{m}) & \;(\xi_{k}\geq \sigma_{m}) \end{array}\right.$$

where

(22) $$\left\{\begin{array}{l} k_{1}(\sigma_{m})=1+\frac{\left(1-k_{0})[-\xi_{t}(2\xi_{c}+\xi_{t})-2\xi_{c}\sigma_{m}+\sigma_{m}^{2}\right]}{{\left(\xi_{c}-\xi_{t}\right)}^{2}}\\ k_{2}(\sigma_{m})=\frac{k_{0}(\bar{\xi }-\sigma_{m})(\bar{\xi }+\sigma_{m}-2\xi_{k})}{{\left(\bar{\xi }-\xi_{k}\right)}^{2}} \end{array}\right.$$

where *ξ_t_*, *ξ_c_* and *ξ_k_* denote the stress region boundaries, as illustrated in Figure 4. According to Han and Chen [37], these parameters take values of 0, *f*_c_/3, and −*f*_c_/3 respectively. The symbol $\bar{\xi }$ represents the intersection point between the yield surface and the hydrostatic axis (see Figure 4).

In summary, the hardening function *k* of SAC can be determined using Equations (21) and (22) as a function of the hardening parameter *k*_0_ and the mean stress *σ*_m_. Once *k* is known, the yield surface of SAC (i.e., *f* = 0) can be constructed via Equation (20). Furthermore, with the yield function explicitly defined, the derivative $\partial f/\partial \sigma_{ij}$ can be calculated using standard tensor differentiation rules. The detailed derivation of this expression is provided in Appendix A.

### 2.4. Effective Stress τ and Stress Scalar Function ϕ

To calibrate the model against the stress–plastic strain response obtained from uniaxial compression tests, the effective stress *τ* and the effective plastic strain increment d*ε_p_* are defined in the context of multiaxial stress states, based on the yield surface function given in Equation (20).

Under uniaxial compression, the principal stress state of the SAC specimen is defined as (*σ*_1_,*σ*_2_,*σ*_3_) = (0,0,−*τ*). Accordingly, the invariants can be expressed as

$$r=\sqrt{\frac{2}{3}}\tau ,\;\sigma_{m}=-\frac{\tau }{3},\;r_{f}=r_{c}(\theta =60^{\circ })$$

Substituting into Equation (20), the yield function simplifies to

(23) $$f=\sqrt{\frac{2}{3}}\tau -kr_{c}=0$$

According to Equation (23), the effective stress can be defined as

(24) $$\tau =\sqrt{\frac{3}{2}}kr_{c}$$

The effective plastic strain increment d*ε_p_* corresponding to the effective stress *τ*, can be derived by first defining the increment of plastic work per unit volume, d*W_p_*, as

(25) $$dW_{p}=\tau d\varepsilon_{p}$$

The plastic work increment per unit volume, d*W_p_*, can alternatively be expressed as

(26) $$dW_{p}=\sigma_{ij}d\varepsilon_{ij}^{p}=\sigma_{ij}d\lambda \frac{\partial g}{\partial \sigma_{ij}}$$

By equating Equations (25) and (26), the following relationship for the effective plastic strain increment is obtained

(27) $$d\varepsilon_{p}=\frac{1}{\tau }\frac{\partial g}{\partial \sigma_{ij}}\sigma_{ij}d\lambda$$

By further equating Equations (27) and (7), the stress scalar function *ϕ* is obtained as

(28) $$\phi =\sqrt{\frac{2}{3}}\frac{\alpha_{p}I_{1}+\sqrt{J_{2}}}{kr_{c}}$$

To derive the expression for ∂f/∂τ, Equation (23) is substituted into Equation (3), which is first reformulated into the following differential form

(29) $$df=\frac{\partial f}{\partial \sigma_{ij}}d\sigma_{ij}+\frac{\partial f}{\partial \tau }d\tau =0$$

Considering the uniaxial compressive stress state, where the only non-zero component of stress tensor *σ_ij_* is *σ*_33_, it follows that d*τ* = −d*σ*_33_ and *σ*_m_ = *σ*_33_/3. Substituting this into Equation (29) yields

(30) $$\frac{\partial f}{\partial \tau }=\frac{\partial f}{\partial \sigma_{33}}$$

Substituting Equation (23) into Equation (30), and noting that both *k* and *r_c_* are functions of the mean stress *σ_m_*, where $\sigma_{m}=\sigma_{33}/3$ under uniaxial compressive state, the following expression of $\partial f/\partial \sigma_{ij}$ is obtained:

(31) $$\frac{\partial f}{\partial \tau }=-(\sqrt{\frac{2}{3}}+\frac{r_{c}}{3}\frac{\partial k}{\partial \sigma_{m}}+\frac{k}{3}\frac{\partial r_{c}}{\partial \sigma_{m}})$$

## 3. Parameter Calibration

To enable the elastoplastic constitutive model developed in Section 2 to accurately represent the multiaxial stress–strain behavior of SAC, it is necessary to calibrate the model parameters and associated variables using experimental data obtained from previously conducted uniaxial and multiaxial mechanical tests. This section details the calibration of three key categories of parameters required for constitutive modeling: the hardening parameters *k*_0_, the plastic modulus *H^p^*, and the plastic volumetric expansion coefficient *α_p_* (see Equation (14)).

### 3.1. Hardening Parameter k_0_

The hardening parameter *k*_0_ can be calibrated by identifying the intersection point between the uniaxial compressive loading path and the loading surface, as illustrated in Figure 3. The calibration procedure is described as follows:

According to experimental results from uniaxial compression tests on SAC [35], the nonlinear segment of the stress–strain curve typically begins when the applied compressive stress reaches approximately −0.5*f_c_* (where compression is taken as negative). Accordingly, this study defines the yield stress of SAC under monotonic uniaxial compression as −0.5*f_c_*.

During monotonic loading, once the uniaxial compressive stress exceeds 0.5*f_c_* in magnitude, the SAC specimen exhibits continuous strain hardening until failure. In this process, the third principal stress *σ*_3_ is the only non-zero principal stress acting on the specimen, which corresponds to the effective stress $\bar{\sigma }$. The mean stress is then given by *σ_m_* = *σ*_3_/3=$-\bar{\sigma }/3$. Notably, this value lies between the boundary parameters *ξ_c_* and *ξ_t_*.

Based on Equations (18)–(22), the following expressions can be obtained:

(32) $$k(k_{0},\sigma_{m})=\frac{r}{r_{f}(\sigma_{m},\theta )}=k_{1}(\sigma_{m})$$

where $r=\sqrt{2J_{2}}=\sqrt{\frac{2}{3}}\bar{\sigma }$, $r_{f}(\sigma_{m},\theta )=r_{c}=\sqrt{3}af_{c}{\left(\frac{b-\frac{\sigma_{m}}{f_{c}}}{c_{c}-\frac{\sigma_{m}}{f_{c}}}\right)}^{d}$.

Solving Equation (32) yields the hardening parameter *k*_0_ as an implicit function of $\bar{\sigma }$:

(33) $$k_{0}=1+\frac{f_{c}^{2}}{{\bar{\sigma }}^{2}-2f_{c}\bar{\sigma }}(1-\frac{r}{{\left.r_{c}\right|}_{\sigma_{m}=-\frac{\bar{\sigma }}{3}}})$$

Substituting $\bar{\sigma }$ = *f_c_* (corresponding to the uniaxial compressive failure state) into Equation (33) yields *k*_0_ = 1.0063 ≈ 1. This result confirms the high accuracy of the proposed formulation in predicting the hardening parameter at ultimate failure. Furthermore, substituting $\bar{\sigma }$ = 0.5 *f_c_* into Equation (33) provides the initial yield parameter as *k*_0_ = *k_y_* = 0.3297.

The initial yield surface function of SAC can then be constructed using the following steps:

(1) Substitute *k*_0_ = 0.3297 and the corresponding mean stress *σ_m_* = −*f_c_*/6 (where compression is considered negative) into Equations (21) and (22) to obtain the hardening function *k*(*k*_0_,*σ_m_*) associated with the initial yield surface.

(2) Substitute the resulting hardening function *k* into Equation (20) to obtain the initial yield surface function.

Similarly, a loading surface (i.e., subsequent yield surface) for SAC can be constructed for any value of *k*_0_ within the continuous range [*k_y_*,1] = [0.3297,1], using the same procedure employed in constructing the initial yield surface.

The calculation method for *k*_0_ based on Equation (33) is strictly applicable to uniaxial compressive loading paths. To extend this approach to multiaxial stress conditions, Equation (33) must be appropriately modified to enable the determination of the hardening parameter $k_{0}^{T}$ under general multiaxial loading. This modification is achieved by replacing the effective stress $\bar{\sigma }$ and the uniaxial compressive failure stress *f_c_* in Equation (33) with their multiaxial counterparts, 3*σ_m_* and 3*σ_mu_*, respectively. The resulting expression is given by:

(34) $$k_{0}^{T}=1-\frac{\sigma_{mu}^{2}}{\sigma_{m}^{2}-2\sigma_{m}\sigma_{mu}}(1-\frac{r}{r_{f}})$$

where *σ_m_* denotes the actual mean compressive stress of the SAC specimen under multiaxial loading, and *σ_mu_* is the predicted mean compressive stress at failure, determined using the Guo–Wang failure criterion (see prediction methodology in Reference [34]).

The final procedure for determining the loading surface function of SAC under multiaxial stress states is outlined as follows:

(1) Substitute the hardening parameter $k_{0}^{T}$ obtained from Equation (34), along with the corresponding mean compressive stress *σ_m_* into Equations (21) and (22) to compute the hardening function *k* under multiaxial loading conditions.

(2) Insert the resulting hardening function *k* into Equation (20) to construct the multiaxial loading surface function of SAC.

### 3.2. Plastic Modulus H^p^

According to Equation (4), *H^p^* represents the tangent modulus of the *τ*-*ε_p_* curve for SAC under complex stress states. However, direct experimental calibration of this relationship is impractical. A feasible approach involves first calibrating the baseline plastic modulus $H_{b}^{p}$ (corresponding to uniaxial compression) using the uniaxial compressive stress–plastic strain curve, and then modifying $H_{b}^{p}$ by introducing a correction coefficient *N*(*σ_m_*,*θ*) that accounts for the combined influence of hydrostatic pressure *σ_m_* and the Lode angle *θ* on the ductility evolution of SAC [39].

(35) $$H^{p}=N(\sigma_{m},\theta )H_{b}^{p}$$

The explicit form of the correction coefficient *N*(*σ_m_*,*θ*) is provided in Equation (39).

**Figure 5:**
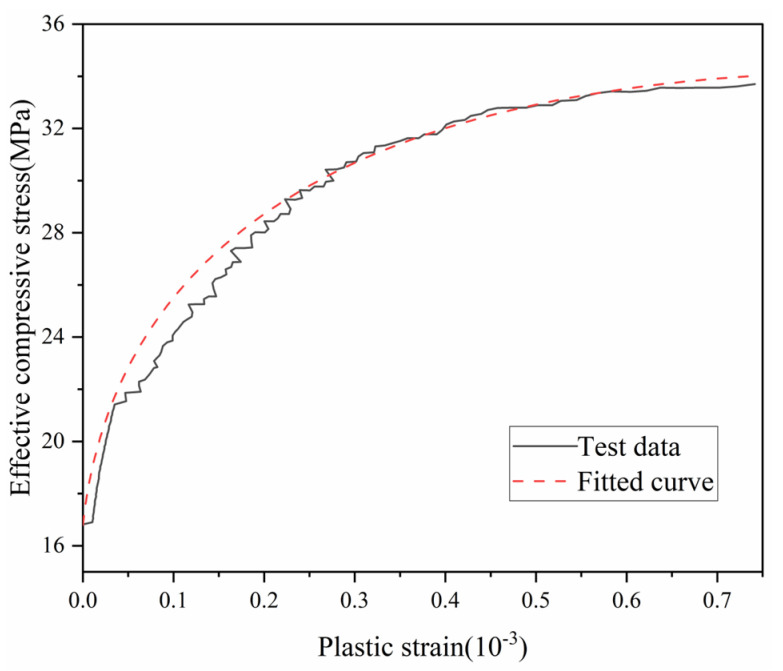
Uniaxial compressive stress versus plastic strain curve of SAC.

The fundamental plastic modulus $H_{b}^{p}$ of SAC can be determined from uniaxial compression tests by treating SAC as an elastoplastic material with an elastic limit of 0.5*f_c_*. The elastic modulus *E* is defined as the tangent modulus at the origin of the uniaxial stress–strain curve. Accordingly, the total strain under uniaxial loading is decomposed into elastic ($\bar{\varepsilon_{e}}$) and plastic ($\bar{\varepsilon_{p}}$) components, with the compressive stress beyond the elastic limit defined as the effective compressive stress $\bar{\sigma }$. Experimental data from uniaxial compression tests [39], plotted in Figure 5 as the $\bar{\sigma }$ − $\bar{\varepsilon_{p}}$ relationship, exhibits apparent discontinuities due to the discrete nature of strain acquisition during testing. This results in non-differentiable, unsmoothed curves that are unsuitable for direct use in constitutive modeling. To address this, the present study employs the functional form defined in Equation (36) to fit the experimental data, with the fitted curve shown as a dashed line in Figure 5.

(36) $$\bar{\varepsilon_{p}}=8.23\times 10^{-5}\times (29.91-\sqrt{827.89-24.22\bar{\sigma }})-\frac{\bar{\sigma }}{22\times 10^{3}}$$

The fundamental plastic modulus $H_{b}^{p}$ of SAC is subsequently derived by differentiating Equation (36) with respect to the plastic strain $\bar{\varepsilon_{p}}$, yielding

(37) $$H_{b}^{p}=\frac{d\bar{\sigma }}{d\bar{\varepsilon_{p}}}=\frac{1}{\frac{d\bar{\varepsilon_{p}}}{d\bar{\sigma }}}=\frac{10^{5}}{4.545-\frac{99.665}{\sqrt{827.89-24.22\bar{\sigma }}}}$$

**Figure 6:**
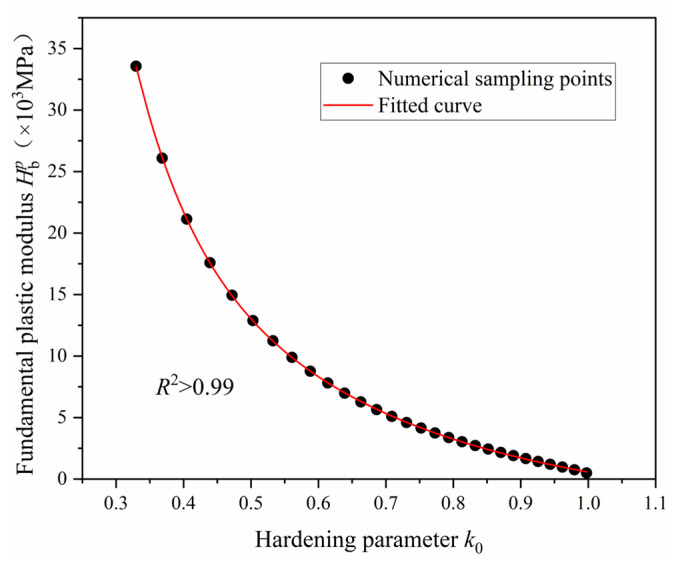
*k*_0_-$H_{b}^{p}$ relationship for SAC.

This expression provides a closed-form representation of $H_{b}^{p}$ as a function of the effective compressive stress $\bar{\sigma }$, enabling direct evaluation during numerical implementation of the constitutive model. While Equation (37) facilitates the calibration of SAC’s fundamental plastic modulus $H_{b}^{p}$ under uniaxial compression, determining the plastic modulus under multiaxial stress states requires establishing an intrinsic relationship between the hardening parameter *k*_0_ and $H_{b}^{p}$. Although the *k*_0_-$H_{b}^{p}$ relationship could, in principle, be derived analytically by solving Equations (33) and (37) simultaneously and eliminating the intermediate variable $\bar{\sigma }$, the complex form of Equation (34) precludes a concise closed-form solution for *k*_0_. Therefore, a practical approach is adopted: for any prescribed stress value ($\bar{\sigma }$)*_i_*, the corresponding values of (*k*_0_)*_i_* and ($H_{b}^{p}$)*_i_* are computed using Equations (33) and (37), respectively. These resulting data pairs ((*k*_0_)*_i_*, ($H_{b}^{p}$)*_i_*) (see Figure 6) define discrete points describing the *k*_0_-$H_{b}^{p}$ relationship. By systematically varying ($\bar{\sigma }$)*_i_* across a representative range, a comprehensive set of data points is generated. The functional form of the *k*_0_-$H_{b}^{p}$ relationship is then established via least-squares curve fitting of these discrete points.

Figure 6 presents the numerical results of the *k*_0_-$H_{b}^{p}$ relationship, where the discrete data points represent the computed pairs ((*k*_0_)*_i_*, ($H_{b}^{p}$)*_i_*). The fitted curve adopts the functional form given in Equation (38), with optimized fitting parameters provided in Table 2. Both Figure 4 and Table 2 confirm that the fitted curve accurately captures the overall trend of the discrete data, yielding a high correlation coefficient (*R*^2^ > 0.99). This strong agreement verifies the validity of the proposed methodology for establishing the *k*_0_-$H_{b}^{p}$ relationship.

(38) $$H_{b}^{p}=A_{1}\exp(-\frac{k_{0}}{t_{1}})+A_{2}\exp(-\frac{k_{0}}{t_{2}})+A_{3}$$

Equation (38) provides an efficient approach for calculating the fundamental plastic modulus $H_{b}^{p}$ of SAC under uniaxial compression. To extend this framework to multiaxial stress states, a correction coefficient *N*(*σ_m_*,*θ*) is introduced and applied to $H_{b}^{p}$. Following the methodology recommended in Reference [40], *N*(*σ_m_*,*θ*) is computed using Equations (39) and (40) as follows:

(39) $$N(\sigma_{m},\theta )=\left\{\begin{array}{ll} P_{m}(\sigma_{m},\theta ) & \;0<P_{m}(\sigma_{m},\theta )\leq 1\\ 1 & \;\mathrm{otherwise} \end{array}\right.$$

where

(40) $$P_{m}(\sigma_{m},\theta )=\frac{-0.15}{\left(1.4-\cos\theta )(\sigma_{m}+\frac{1}{3})(\sigma_{m}+2.5\right)}$$

### 3.3. Coefficient of Cubical Expansion α_p_

As defined in Equation (16), *α_p_* characterizes the plastic volumetric expansion of SAC under external loading. Given the intrinsic correlation between the evolution of plastic deformation and the material’s hardening state, *α_p_* can be calibrated using the hardening parameter *k*_0_. Following the methodology proposed in Reference [41], this study assumes a linear relationship between *α_p_* and *k*_0_:

(41) $$\alpha_{p}=\left\{\begin{array}{ll} (\alpha_{L}-\alpha_{U})\frac{1-k_{0}}{1-k_{y}}+\alpha_{U} & \;\mathrm{for}\;I_{1}<0\\ 0 & \;\mathrm{for}\;I_{1}>0 \end{array}\right.$$

where values of *α_L_* and *α_U_* are determined by the parameter ψ (with ψ = *σ*_max_/*σ*_min_). Their computation follows:

(42) $$\begin{array}{ll} \mathrm{for}\;\psi <0 & \;\alpha_{L}=\alpha_{1}(1+\psi ),\;\alpha_{U}=\alpha_{2}(1+\psi )\\ \mathrm{for}\;0<\psi <0.1 & \;\alpha_{L}=\alpha_{1}-(0.8+\alpha_{1})\psi ,\;\alpha_{U}=\alpha_{2}+10(0.1-\alpha_{2})\psi \\ \mathrm{for}\;\psi >0.1 & \;\alpha_{L}=\alpha_{1}-(0.8+\alpha_{1})\psi ,\;\alpha_{U}=0.1 \end{array}$$

where *α*_1_ and *α*_2_ are material constants representing the strength characteristics of concrete, assigned values of −0.6 and 0.2, respectively.

## 4. Numerical Calculation and Model Verification

The elastoplastic constitutive model for SAC developed in Section 2 and Section 3 theoretically characterizes the incremental stress–strain relationship between infinitesimal stress increments (d*σ_ij_*) and infinitesimal strain increments (d*ε_ij_*) under a given stress or plastic deformation history. However, in numerical simulations of practical engineering problems, each loading step typically involves finite increments of stress (Δ*σ*) or strain (Δ*ε*). As a result, the incremental constitutive formulation alone is insufficient to directly capture the stress–strain response of SAC under multiaxial stress states, thereby necessitating numerical integration schemes for model implementation and calibration.

### 4.1. Numerical Calculation Methodology

#### 4.1.1. Matrix-Form Multiaxial Stress–Strain Relationship

The tensor-form constitutive relations of SAC are explicitly converted into matrix notation as follows. To begin with, the generalized Hooke’s law in matrix form is written as:

(43) $$\begin{array}{l} \left\{d\sigma \right\}=\left[C\right]\left\{d\varepsilon^{e}\right\}=\left[C\right](\left\{d\varepsilon \right\}-\left\{d\varepsilon^{p}\right\})\\ \mathrm{or}\;\;\left\{d\sigma \right\}=\left[C^{eq}\right]\left\{d\varepsilon \right\} \end{array}$$

where {d*σ*} and {d*ε*} denote the stress and total strain increment vectors, respectively. The matrices [*C*] and [*C^eq^*] represent the elastic stiffness matrix and the elastoplastic stiffness matrix of steel slag aggregate concrete (SAC), respectively. The vector {d*ε^e^*} and {d*ε^p^*} correspond to the elastic and plastic strain increments. All vectors adopt Voigt notation (6 × 1), and all matrices are of size 6 × 6. The explicit form of the elastic stiffness matrix [*C*] is presented as follows:

(44) $$\left[C\right]=\frac{E}{\left(1+\nu )(1-2\nu \right)}\left[\begin{array}{cccccc} 1-\nu & \nu & \nu & 0 & 0 & 0\\ \nu & 1-\nu & \nu & 0 & 0 & 0\\ \nu & \nu & 1-\nu & 0 & 0 & 0\\ 0 & 0 & 0 & \frac{1-2\nu }{2} & 0 & 0\\ 0 & 0 & 0 & 0 & \frac{1-2\nu }{2} & 0\\ 0 & 0 & 0 & 0 & 0 & \frac{1-2\nu }{2} \end{array}\right]$$

where *E* represents the uniaxial elastic modulus and *ν* denotes Poisson’s ratio. Based on uniaxial compression tests of SAC [35], the parameters are determined as *E* = 3.05 × 104 MPa and *ν* = 0.226.

The plastic strain increment in Equation (43) is expressed in matrix form based on the non-associated flow rule as

(45) $$\left\{d\varepsilon^{p}\right\}=d\lambda \left\{\frac{\partial g}{\partial \left\{\sigma \right\}}\right\}$$

where the functional form of *g* is specified in Equation (14), and the non-negative scalar d*λ* is given by

(46) $$d\lambda =\frac{L}{h}$$

Here, the scalar function *h* is computed according to Equation (9), and the loading criterion function *L* is defined by the following matrix expression

(47) $$L=\left\{\frac{\partial f}{\partial \left\{\sigma \right\}}\right\}\left[C\right]\left\{d\varepsilon \right\}$$

where *f* = *f* ({*σ*},*κ*) denotes the yield function of SAC, *κ* is the hardening parameter, and *κ* = *k*_0_ in this study.

The elastoplastic stiffness matrix in Equation (43) can then be expressed as

(48) $$\left[C^{eq}\right]=\left[C\right]-\frac{1}{h}{\left[H^{*}\right]}^{T}\left[H\right]$$

where

(49) $$\left[H\right]=\left[H_{xx},H_{yy},H_{zz},H_{yz},H_{xz},H_{xy}\right]$$

(50) $$\left[H^{*}\right]=\left[H_{xx}^{*},H_{yy}^{*},H_{zz}^{*},H_{yz}^{*},H_{xz}^{*},H_{xy}^{*}\right]$$

The elements of matrices $\left[H\right]$ and $\left[H^{*}\right]$ are computed according to Equations (13) and (12), respectively.

#### 4.1.2. Analysis of the Numerical Computation Procedure

The numerical implementation involves computing the stress state *^n^*^+1^{*σ*} at step (n + 1), based on the known stress *^n^*{*σ*} and strain *^n^*{*ε*} after *n* strain-controlled loading steps. To determine *^n^*^+1^{*σ*}, two key procedures are required: first, identifying the material state at step n (i.e., elastic or inelastic) to select either the linear elastic formulation or the elastoplastic constitutive model defined in Equation (43); second, applying an appropriate numerical integration scheme consistent with the identified material response. Accordingly, two scenarios are considered for the current state: (1) elastic, or (2) elastoplastic.

1. Loading State Analysis

(1) The specimen is currently in an elastic state

If the specimen is currently in an elastic state, the SAC initial yield function satisfies f({σ}n,ky)<0. Upon applying the strain increment $\left\{\Delta \varepsilon \right\}$ at loading step (n + 1), two potential states emerge: elastic or elastoplastic. Assuming the specimen remains elastic after the increment, the stress increment vector for this step is expressed as follows:

(51) $$\left\{\Delta \sigma \right\}=\left\{\Delta \sigma^{e}\right\}=\left[C\right]\left\{\Delta \varepsilon \right\}$$

where $\left\{\Delta \sigma^{e}\right\}$ denotes the trial elastic stress increment vector.

If f({σ}n+Δσe,ky)≤0, the initial elasticity assumption holds, and the stress and strain states of the specimen are updated as follows:

(52) {σ}n+1={σ}n+Δσe{ε}n+1={ε}n+Δε

If f({σ}n+Δσe,ky)>0, the initial elasticity assumption is invalidated, indicating that the specimen transitions to an elastoplastic state upon applying the strain increment $\left\{\Delta \varepsilon \right\}$ at step (*n* + 1). Given that the specimen was initially elastic prior to this loading step, there must exist a scaling factor *r* (0 < *r* < 1) such that

(53) f({σ}n+rΔσe,ky)=0

Then the stress increment vector $\left\{\Delta \sigma \right\}$ for this loading step is then given by

(54) Δσ=∫{ε}n{ε}+r{Δε}nCdε+∫{ε}+r{Δε}n{ε}+{Δε}nC(dε−dεp)=rΔσe+∫{ε}+r{Δε}n{ε}+{Δε}nC(dε−dεp)=rΔσe+∫{ε}+r{Δε}n{ε}+{Δε}nCeqdε

Finally, the stress state of the specimen is updated as follows:

(55) {σ}n+1={σ}n+Δσ

Notably, updating the stress state via Equation (55) requires determination of the scaling factor *r*. Due to the complex functional form of the SAC yield surface established in Section 2.3, Equation (53) constitutes a higher-order nonlinear equation in *r*, rendering direct analytical solution intractable. To obtain a numerical solution for *r*, a first-order Taylor series expansion of Equation (53) is employed, yielding:

(56) f({σ}n+rΔσe,ky)=f({σ}n,ky)+∂f∂σ{σ}nrΔσe=0

By solving Equation (56), the numerical solution for *r* is conveniently obtained as follows

(57) r=−f({σ}n,κn)∂f∂σ{σ}nΔσe

(2) The specimen is currently in an elastoplastic state

If the specimen is in an elastoplastic state after the *n*-th loading step (characterized by f({σ}n,ky)≥0) and the subsequent yield surface f({σ}n,κn)=0, the loading state during the (n + 1)-th increment {Δ*ε*} must be determined prior to the stress increment {Δ*σ*} computation. Assuming the strain increment {Δ*ε*} is sufficiently small, the loading criterion *L* in Equation (47) is reformulated as follows

(58) $$L=\left\{\frac{\partial f}{\partial \left\{\sigma \right\}}\right\}\left[C\right]\left\{\Delta \varepsilon \right\}$$

If *L* ≤ 0, the elastic boundary of SAC remains unchanged during this loading step, indicating either unloading or neutral loading. The stress increment is computed using Equation (51), and the stress state is updated according to Equation (52).

If *L* > 0, the elastic boundary expands, signifying plastic loading. The stress increment is calculated via Equation (54) (note: *r* = 0 in this case), and the stress state is updated following Equation (55).

It should be noted that due to the monotonic loading protocol employed in the multiaxial mechanical tests [34], once a loading step triggers the elastoplastic state, all subsequent steps remain in the loading state (*L* ≥ 0 and *r* = 0).

2. Numerical integration method

To compute the stress increment {Δ*σ*} from step *n* to (*n* + 1), numerical integration of Equation (54) is required. In this study, the explicit forward Euler algorithm is adopted for this purpose. To enhance computational accuracy, the elastoplastic strain increment (1 − *r*){Δ*ε*} in Equation (54) is discretized into *m* equal substeps $\left\{\Delta \bar{\varepsilon }\right\}$, such that

(59) $$\left\{\Delta \bar{\varepsilon }\right\}=(1-r)\left\{\Delta \varepsilon \right\}/m$$

where *m* is a positive integer greater than 1.

Let {σ}in, {ε}in, and κin denote the stress state, strain state, and hardening parameter, respectively, after applying the *i*-th strain sub-increment $\left\{\Delta \bar{\varepsilon }\right\}$ within the (*n* + 1)-th loading step (1 ≤ *i* ≤ *m*). The stress sub-increment $\left\{\Delta \bar{\sigma }\right\}$ following the (*i* + 1)-th sub-increment $\left\{\Delta \bar{\varepsilon }\right\}$ is computed using Equation (60) or (61), based on Equation (43):

(60) $$\left\{\Delta \bar{\sigma }\right\}=\left[C\right](\left\{\Delta \bar{\varepsilon }\right\}-\left\{\Delta {\bar{\varepsilon }}^{p}\right\})$$

(61) $$\left\{\Delta \bar{\sigma }\right\}=\left[C^{eq}\right]\left\{\Delta \bar{\varepsilon }\right\}$$

where $\left\{\Delta {\bar{\varepsilon }}^{p}\right\}$ denotes the plastic component of the strain sub-increment $\left\{\Delta \bar{\varepsilon }\right\}$, and the elastoplastic stiffness matrix $\left[C^{eq}\right]$ is determined according to Equation (48). Based on Equations (46) and (47), $\left\{\Delta {\bar{\varepsilon }}^{p}\right\}$ is computed as

(62) $$\left\{\Delta {\bar{\varepsilon }}^{p}\right\}=\left[P\right]\left\{\Delta \bar{\varepsilon }\right\}$$

where

(63) P=P(σ,κin)=1h∂g∂σ∂f∂σTC

with g=g(σ,κin), f=f(σ,κin), the scalar function *h* evaluated using Equation (9).

Upon completion of the (*i* + 1)-th strain sub-increment $\left\{\Delta \bar{\varepsilon }\right\}$, the stress state of the SAC specimen is updated as follows

(64) {σ}i+1n ={σ{in+Δσ¯

Using the updated stress state {σ}i+1n , the hardening parameter *k*_0_ (denoted as κi+1n ) is updated according to the methodology described in Section 3.1. With {σ}i+1n  and κi+1n  obtained after the (*i* + 1)-th strain sub-increment $\left\{\Delta \bar{\varepsilon }\right\}$, the computational cycle defined by Equations (60) to (64) is iteratively executed to update stress and hardening parameters for subsequent sub-increments. Ultimately, after *m* iterations, the stress state {σ}n+1 at the end of the (*n* + 1)-th loading step is obtained.

Finally, it should be noted that, according to Equations (62) and (63), the forward Euler algorithm utilizes the yield surface *f* and plastic potential function *g* from the previous sub-increment to compute the plastic strain increment $\left\{\Delta {\bar{\varepsilon }}^{p}\right\}$ for the current sub-increment. Since both the yield surface and plastic potential evolve continuously during the application of $\left\{\Delta \bar{\varepsilon }\right\}$, this lagged approximation inevitably introduces numerical errors. To mitigate such errors, the number of substeps *m* should be sufficiently increased to minimize variations in *f* and *g* within each sub-increment, thereby reducing the resulting numerical inaccuracies to negligible levels.

### 4.2. Numerical Implementation and Validation Results of the Constitutive Model

**Figure 7:**
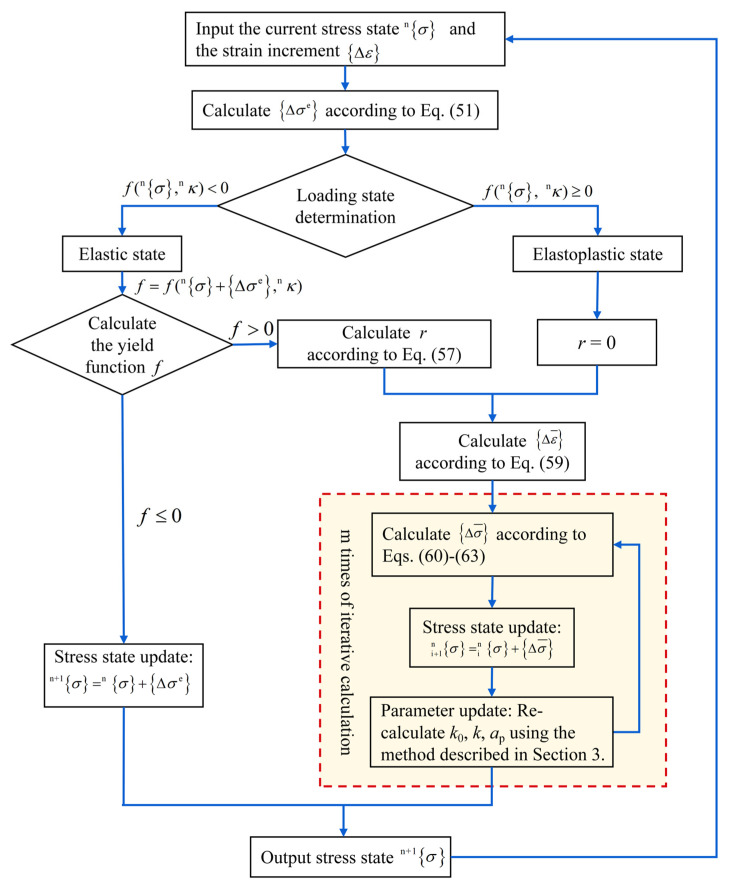
Computational flowchart of the SAC elastoplastic constitutive model.

Figure 7 illustrates the computational flowchart of the SAC elastoplastic constitutive model based on the numerical methodology presented in Section 4.1. The flowchart delineates three distinct regimes. Initially, when the applied stress state remains within the initial yield surface, the material response is classified as elastic or neutral, and the output stress is computed by directly summing the input stress and the trial elastic stress increment $\left\{\Delta \sigma^{e}\right\}$. Once a critical loading step is reached and the stress state crosses the initial yield surface (indicating a transition to the elastoplastic regime), the scaling factor *r* is computed to identify the precise yield point, followed by numerical integration of the plastic sub-increment. Subsequently, under continued monotonic loading, all following steps assume *r* = 0 (i.e., immediate yielding), and stresses are updated through the elastoplastic integration cycle.

**Figure 8:**
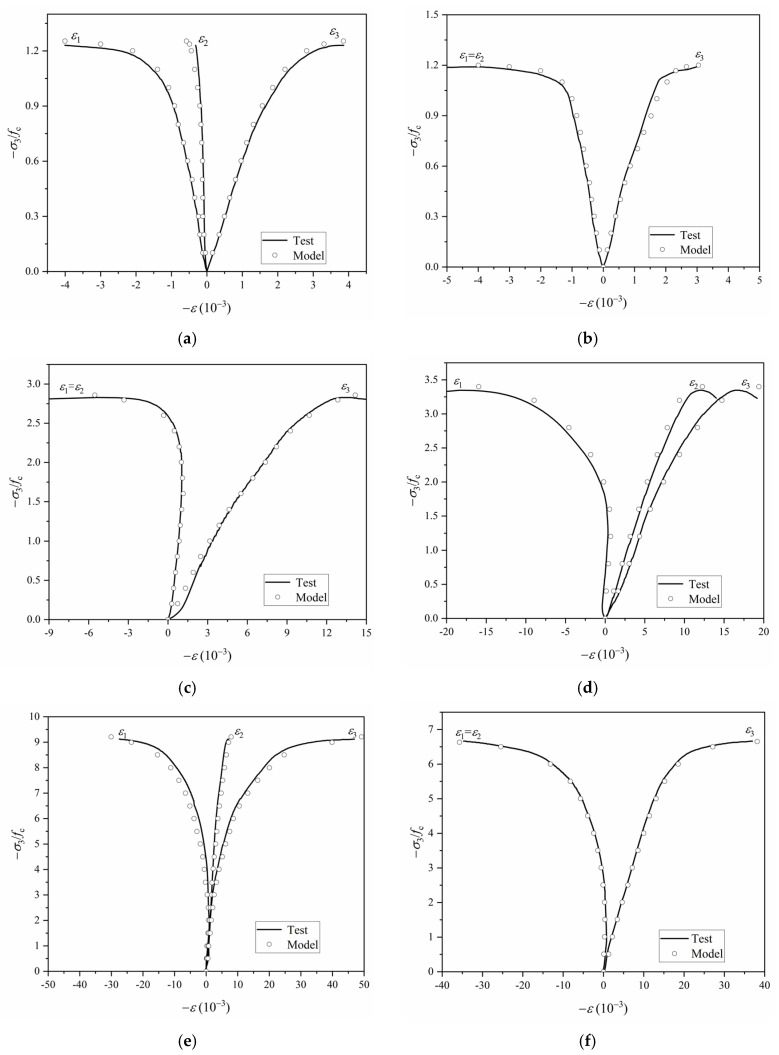
Validation results of the SAC elastoplastic constitutive model. (**a**) Biaxial compression (*σ*_2_:*σ*_3_ = −0.25:−1); (**b**) Biaxial compression (*σ*_2_:*σ*_3_ = −1:−1); (**c**) Triaxial compression (*σ*_1_:*σ*_2_:*σ*_3_ = −0.1:−0.1:−1); (**d**) Triaxial compression (*σ*_1_:*σ*_2_:*σ*_3_ = −0.1:−0.75:−1); (**e**) Triaxial compression (*σ*_1_:*σ*_2_:*σ*_3_ = −0.2:−0.5:−1); (**f**) Triaxial compression (*σ*_1_:*σ*_2_:*σ*_3_ = −0.2:−1:−1).

Following the computational flowchart illustrated in Figure 7, a MATLAB program was developed to simulate the stress–strain responses of SAC specimens under three representative stress states: biaxial compression (*σ*_1_:*σ*_3_ = 0), and triaxial compression with stress ratios of *σ*_1_:*σ*_3_ = 0.1 and *σ*_1_:*σ*_3_ = 0.2, respectively. The theoretical predictions were subsequently validated against experimental data reported in [34]. To ensure numerical convergence and assess sensitivity, five substep configurations were adopted for each simulation (*m* = 10^1^, 10^2^, 10^3^, 10^4^, 10^5^). A comparative analysis of the numerical and experimental results is presented in Figure 8.

The comparative results presented in Figure 8 demonstrate excellent agreement between the theoretical predictions and experimental measurements under proportional monotonic loading conditions. Notably, the predictive accuracy of the proposed constitutive model remains robust across different stress ratios, confirming its capability to reliably capture the stress–strain behavior of SAC specimens subjected to multiaxial stress states with arbitrary stress ratios.

## 5. Conclusions

To support the engineering application of steel slag aggregate concrete (SAC), this study developed an elastoplastic constitutive model for SAC based on the non-uniform hardening plasticity model proposed by Chen et al. [38], within the framework of classical plasticity theory. The model was rigorously validated against both uniaxial and multiaxial mechanical test data of SAC. The main conclusions are summarized as follows:

(1) A failure surface and corresponding yield criterion for SAC were formulated based on the Guo–Wang failure criterion. This yield function effectively characterizes the distinct mechanical responses of SAC across various stress regimes, including brittle failure under tensile stress, enhanced ductility under high confining pressure, and pronounced sensitivity to hydrostatic pressure.

(2) A multiaxial incremental elastoplastic stress–strain relationship for SAC was derived using classical plasticity theory. The formulation employs a non-associated flow rule with a Drucker–Prager-type plastic potential function and satisfies the consistency condition. A parameter calibration procedure was established to determine the hardening parameter *k*_0_ and plastic modulus *H^p^*, utilizing both uniaxial and multiaxial test data.

(3) The computational procedure for evaluating stress increments during elastoplastic deformation was detailed, and a numerical algorithm tailored to the proposed constitutive model was developed. This algorithm was implemented in MATLAB for practical simulation.

(4) The developed model was validated through numerical simulations using MATLAB and corresponding multiaxial test data of SAC. The results demonstrate that the model accurately reproduces the stress–strain responses of SAC under various loading conditions, including biaxial compression (*σ*_1_:*σ*_3_ = 0), triaxial compression with low confining pressure (*σ*_1_:*σ*_3_ = 0.1), and triaxial compression with high confining pressure (*σ*_1_:*σ*_3_ = 0.2).

In future work, the proposed multiaxial constitutive model will be extended to engineering applications, including structural design and performance prediction of SAC under complex loading conditions. Particular attention will be given to durability under long-term multiaxial stresses and the response of heavy-load infrastructures such as pavements and foundations.

## Figures and Tables

**Table 1:** Parameters of SAC failure criterion [34].

Parameters	*a*	*b*	*c_t_*	*c_c_*	*d*	*α*	*β*
Value	10.0632	0.0976	17.8701	11.0764	0.9326	1.6	2.0

**Table 2:** Parameters of *k*_0_-$H_{b}^{p}$ relationship.

Parameters	*A*_1_	*A*_2_	*A*_3_	*t*_1_	*t*_2_	Correlation Coefficient *R*^2^
Value	6.67 × 10^6^	5.60 × 10^5^	−2.99 × 10^4^	0.0852	0.3632	>0.99

## Data Availability

The original contributions presented in this study are included in the article. Further inquiries can be directed to the corresponding authors.

## Notation

*σ*_1_, *σ*_2_, *σ*_3_	Principal stresses (tension-positive)
*σ_ij_*	Stress tensor
*s_ij_*	Deviatoric stress tensor
*σ*_m_	Mean normal stress
*τ*_m_	Mean shear stress
*ε*_1_, *ε*_2_, *ε*_3_	Principal strain
*ε_ij_*	Strain tensor
*ε*_v_	Volumetric strain
*I*_1_ = *σ_kk_*	The first invariant of the stress tensor
$J_{2}=\frac{1}{2}s_{ij}s_{ij}$	The second invariant of the deviatoric stress tensor
$J_{3}=\frac{1}{3}s_{ij}s_{jk}s_{ki}$	The third invariant of the deviatoric stress tensor
*θ*	Lode angle
$r=\sqrt{2J_{2}}$	Length of the deviatoric part of the stress tensor
*E*	Elasticity modulus
*v*	Poisson’s ratio
$C_{ijkl}^{e}$	Elastic stiffness tensor
$C_{ijkl}^{eq}$	Elastoplastic stiffness tensor
$\delta_{ij}$	Permutation operator (Kronecker symbol)
{ }	Vector
[ ]	Matrix

## Appendix A

To facilitate the computation of $\frac{\partial f}{\partial \sigma_{ij}}$, the SAC yield function (Equation (20)) is reformulated as follows:

(A1) $$f=f(I_{1},J_{2},J_{3})=0$$

Then, we have

(A2) $$\frac{\partial f}{\partial \sigma_{ij}}=\frac{\partial f}{\partial I_{1}}\frac{\partial I_{1}}{\partial \sigma_{ij}}+\frac{\partial f}{\partial J_{2}}\frac{\partial J_{2}}{\partial \sigma_{ij}}+\frac{\partial f}{\partial J_{3}}\frac{\partial J_{3}}{\partial \sigma_{ij}}$$

By applying tensor differentiation rules, the following relationship holds:

(A3) $$\begin{array}{l} \frac{\partial I_{1}}{\partial \sigma_{ij}}=\delta_{ij}\\ \frac{\partial J_{2}}{\partial \sigma_{ij}}=s_{ij}\\ \frac{\partial J_{3}}{\partial \sigma_{ij}}=s_{ik}s_{kj}-\frac{2}{3}J_{2}\delta_{ij}=t_{ij} \end{array}$$

Substituting Equation (A3) into Equation (A2) and assigning $B_{0}=\frac{\partial f}{\partial I_{1}},B_{1}=\frac{\partial f}{\partial J_{2}},B_{2}=\frac{\partial f}{\partial J_{3}}$, thereby obtains

(A4) $$\frac{\partial f}{\partial \sigma_{ij}}=B_{0}\delta_{ij}+B_{1}s_{ij}+B_{2}t_{ij}$$

Rewriting Equation (20) in simplified form as *f* = *r* − *kr_f_*, and substituting this expression into *B*_0_, *B*_1_, and *B*_2_ yields:

(A5) $$\begin{array}{l} B_{0}=\frac{\partial f}{\partial I_{1}}=-\frac{\partial k}{\partial I_{1}}r_{f}-k\frac{\partial r_{f}}{\partial I_{1}}\\ B_{1}=\frac{\partial f}{\partial J_{2}}=\frac{1}{r}-k\frac{\partial r_{f}}{\partial J_{2}}\\ B_{2}=\frac{\partial f}{\partial J_{3}}=-k\frac{\partial r_{f}}{\partial J_{3}} \end{array}$$

To facilitate solving for $\frac{\partial r_{f}}{\partial I_{1}},\frac{\partial r_{f}}{\partial J_{2}},\frac{\partial r_{f}}{\partial J_{3}}$, Equation (18) is rewritten in the following simplified form:

(A6) $$r_{f}=\sqrt{3}af_{c}{\left(\frac{s}{t}\right)}^{d}$$

where

(A7) $$\begin{array}{l} s=s(\sigma_{m})=b-\frac{\sigma_{m}}{f_{c}}\\ t=t(\sigma_{m},\theta )=c-\frac{\sigma_{m}}{f_{c}} \end{array}$$

Based on Equations (A6) and (A7), the expressions for $\frac{\partial r_{f}}{\partial I_{1}},\frac{\partial r_{f}}{\partial J_{2}},\frac{\partial r_{f}}{\partial J_{3}}$ are derived as follows:

(A8) $$\begin{array}{l} \frac{\partial r_{f}}{\partial I_{1}}=\sqrt{3}adf_{c}{\left(\frac{s}{t}\right)}^{d-1}(\frac{\frac{\partial s}{\partial I_{1}}t-\frac{\partial t}{\partial I_{1}}s}{t^{2}})\\ \frac{\partial r_{f}}{\partial J_{2}}=\sqrt{3}adf_{c}{\left(\frac{s}{t}\right)}^{d-1}(\frac{\frac{\partial s}{\partial J_{2}}t-\frac{\partial t}{\partial J_{2}}s}{t^{2}})\\ \frac{\partial r_{f}}{\partial J_{3}}=\sqrt{3}adf_{c}{\left(\frac{s}{t}\right)}^{d-1}(\frac{\frac{\partial s}{\partial J_{3}}t-\frac{\partial t}{\partial J_{3}}s}{t^{2}}) \end{array}$$

Taking the partial derivative of Equation (A7) yields

(A9) $$\begin{array}{l} \frac{\partial s}{\partial I_{1}}=\frac{\partial (b-\frac{\sigma_{m}}{f_{c}})}{\partial I_{1}}=-\frac{1}{f_{c}}\frac{\partial \sigma_{m}}{\partial I_{1}}=-\frac{1}{f_{c}}\frac{\partial (\frac{1}{3}\sigma_{kk})}{\partial \sigma_{kk}}=-\frac{1}{3f_{c}}\\ \frac{\partial t}{\partial I_{1}}=\frac{\partial (c-\frac{\sigma_{m}}{f_{c}})}{\partial I_{1}}=\frac{\partial c}{\partial I_{1}}-\frac{1}{3f_{c}}=\frac{\partial c}{\partial \theta }\frac{\partial \theta }{\partial I_{1}}--\frac{1}{3f_{c}}\\ \frac{\partial t}{\partial J_{2}}=\frac{\partial (c-\frac{\sigma_{m}}{f_{c}})}{\partial J_{2}}=\frac{\partial c}{\partial J_{2}}=\frac{\partial c}{\partial \theta }\frac{\partial \theta }{\partial J_{2}}\\ \frac{\partial t}{\partial J_{3}}=\frac{\partial (c-\frac{\sigma_{m}}{f_{c}})}{\partial J_{3}}=\frac{\partial c}{\partial J_{3}}=\frac{\partial c}{\partial \theta }\frac{\partial \theta }{\partial J_{3}}\\ \frac{\partial s}{\partial J_{2}}=\frac{\partial s}{\partial J_{3}}=0 \end{array}$$

Differentiating Equation (19) with respect to *θ* yields

(A10) $$\frac{\partial c}{\partial \theta }=-1.5\alpha c_{t}(\sin1.5\theta ){\left(\cos1.5\theta \right)}^{\alpha -1}+1.5\beta c_{c}(\cos1.5\theta ){\left(\sin1.5\theta \right)}^{\beta -1}$$

As is well-established; the Lode angle *θ* satisfies the following expression:

(A11) $$\theta =\frac{1}{3}\arccos(\frac{3\sqrt{3}}{2}\frac{J_{3}}{J_{2}^{3/2}})$$

Based on Equation (A11), the partial derivatives of the Lode angle *θ* with respect to the first stress invariant *I*_1_, the second deviatoric invariant *J*_2_, and the third deviatoric invariant *J*_3_ are derived as follows:

(A12) $$\begin{array}{l} \frac{\partial \theta }{\partial I_{1}}=0\\ \frac{\partial \theta }{\partial J_{2}}=\frac{\sqrt{3}J_{3}}{4\sqrt{1-\xi^{2}}}J_{2}^{-5/2}\\ \frac{\partial \theta }{\partial J_{3}}=\frac{1}{2\sqrt{1-\xi^{2}}}J_{2}^{-3/2} \end{array}$$

where $\xi =\frac{3\sqrt{3}}{2}\frac{J_{3}}{J_{2}^{3/2}}$.

In summary, the derivation demonstrates that intermediate parameters *B*_0_, *B*_1_, and *B*_2_ are obtained via Equations (A5) to (A12). Substituting these intermediate parameters into Equation (A4) ultimately yields $\frac{\partial f}{\partial \sigma_{ij}}$.
